# Everyday life for patients with schizophrenia and severely impaired social functioning

**DOI:** 10.3389/fpsyt.2024.1399935

**Published:** 2024-06-05

**Authors:** Nanna Yosser Ben Høier, Ida-Marie Mølstrøm, Annick Urfer-Parnas, Mads Gram Henriksen, Julie Nordgaard

**Affiliations:** ^1^ Mental Health Center Amager, Copenhagen University Hospital, Copenhagen, Denmark; ^2^ Department of Clinical Medicine, Faculty of Health and Medical Science, University of Copenhagen, Copenhagen, Denmark; ^3^ Center for Subjectivity Research, Department of Communication, University of Copenhagen, Copenhagen, Denmark

**Keywords:** sociality, homelessness, structuring elements, positive withdrawal, schizophrenia

## Abstract

**Background:**

A structure of everyday life creates routines and a sense of familiarity, which provides a recognizable basis for being and acting in the world. A structure of everyday life reduces stress, and daily stress has consistently been associated with higher levels of psychiatric symptoms. Little is known about how patients with schizophrenia and severe social impairment structure their lives. Thus, we aimed to explore the everyday lives of this group of patients, looking for structuring elements.

**Methods:**

In this qualitative study, we included patients diagnosed with schizophrenia who were either homeless or had difficulties reporting for treatment and, thus, needed treatment from an outreach team.

**Results:**

17 participants were included in the study. We found only few structuring elements across all participants in the qualitative analyses. We identified five themes in our sample that serve as structuring elements in the everyday life: social interactions, volunteering to assist with basic tasks, self-initiated routines such as going for a walk daily, exoskeleton (structure provided by others), and having pets. None of the participants reported much activity during the day, and for most of them, social interactions were minimal.

**Discussion:**

All the participants had very little structure and routines in their everyday life. The shelters provided the homeless participants with some structuring elements, whereas the domiciled participants had no external structuring elements. The findings have important implications for psychosocial treatment of severe social impairment in schizophrenia. The lack of structure in these patients’ everyday lives highlights the need for targeted interventions that could facilitate such structures and guide social involvement and personal recovery.

## Introduction

Difficulties in social functioning are well-known features of schizophrenia, and they tend to precede the onset of psychosis and continue after symptomatic remission (1–3). Social functioning is considered a key component of recovery, and some argue that good social functioning is more important than remission from psychotic symptoms (4). Consequently, improving social functioning has been a target of many therapeutic approaches, including psychosocial rehabilitation and recovery-orientated work, showing varying results and effects, especially in the poor outcome group (5), which amounts to approximately—40% of patients with schizophrenia (6).

One aspect of social functioning is creating and continuing some routines of everyday life, which reduces the number of decisions that need to be considered every day and gives a sense of familiarity which provides us with a consistent and persistent way of being and acting in the world. Such a structure of everyday life reduces stress (7), and it has consistently been shown that daily stressors are associated with higher levels of psychiatric symptoms (8).

The silent background of everyday life, with its daily rhythm and automaticity, has been reported to be particularly precarious in some patients with schizophrenia (5). Schizophrenia, Troubé argued, may affect the rules and structures governing ‘everydayness,’ potentially depriving the everydayness of its usual sense of familiarity, reliability, and predictability (9).

Research into social functioning and quality of life has been ongoing for several decades. However, the focus has mostly been on quantitative studies, where social functioning and quality of life typically have been examined as outcome measures (10). This research field is challenged by lack of consensus about definitions, use of various scales, and inconsistent results across studies (3). Qualitative studies on subjective experiences of everyday life by individuals with severe schizophrenia are, sparse (11). Kasén & Bondas (2022) provided a phenomenological-hermeneutic study on the perspectives and experiences of the sufferings in daily living with severe schizophrenia. They found themes oriented around a simultaneous struggle to grasp the illness as well as reshaping the future and reconciling with the losses from illness (12). Avieli et al. (2016) found in their qualitative study among 60–69-year-olds with schizophrenia nine dimensions of suffering, including rejection, dealing with symptoms and side effects, and a loss of social life and hope to be a partner or a parent. They concluded that the severity and impact on quality of life and social function in schizophrenia do not reduce over time but evoke issues such as existential loneliness (13). Finally, Nilsson et al. (2019) (14) proposed in their qualitative study that impairment in social functioning may reflect compensatory mechanisms, which could be a target for developing new psychotherapeutic interventions.

Previously, psychiatric asylums or institutions provided the daily structure and routine for many patients with schizophrenia. Due to changes in psychiatry and society in general, most patients with schizophrenia today live outside of institutions, leaving the responsibility for structuring everyday life mostly to the patients themselves. For example, Sisti et al. reported that a 95% decline in the per capita number of state psychiatric beds since 1955 in the US (15). Furthermore, they describe how the deinstitutionalization has led to a “transinstitutionalization” of patients into prisons, homeless shelters, and emergency rooms, often in a combination of the three in a recurrent and unstable circle. Others have argued that more factors contribute to the status quo in the care of severe mental illness, including inadequate community care (16).

Given the importance of social functioning in everyday life for recovery, knowledge is needed about how patients with severely impairing schizophrenia structure or do not structure their everyday lives. The purpose of this study was to explore the everyday life of these patients, aiming to achieve a better understanding of the lives lived by individuals diagnosed with schizophrenia who have severely impaired social functioning. Such an understanding could be crucial for developing effective treatment and intervention strategies tailored to the patient’s unique situation and in which they can participate.

## Methods

We explored everyday life in two groups of severely impaired patients with schizophrenia: one group of patients was homeless, living in a shelter, and the other group was domiciled but had substantial difficulties meeting basic social demands such as regularly attending appointments at the outpatient clinic or performing daily chores. Our research questions were as follows:

1. What is a typical day like?
2. What structuring elements do the patients have in their everyday lives?

### Participants and setting

The study was carried out at the Mental Health Center Amager, a general psychiatric hospital in Copenhagen. The study included patients from two psychiatric outreach teams. The first team was the Homeless Outreach Psychiatric Service (HOPS) which seeks out homeless individuals with suspected psychosis in Copenhagen and offers psychiatric evaluation and treatment (17). The second outreach team was a psychiatric outpatient clinic for severe mental illness in Copenhagen, organized as Flexible Assertive Community Treatment (F-ACT) Teams.

The inclusion criteria were a diagnosis of schizophrenia, age between 18 and 65, capability of participating in the interview, and either being homeless or being in a condition for which it is necessary for psychiatric services to visit the patients in their homes, because they are unable to show up regularly at the psychiatric outpatient clinic (F-ACT).

The exclusion criteria were current employment, current hospitalization, and forensic status. All participants received treatment in one of the two psychiatric outreach teams. Treatment varied but consisted of elements of antipsychotic medication, social support, psychological support, psychoeducation, and peer support.

All participants were able to receive written and verbal information about the study and were evaluated as eligible for participation by their outpatient psychiatrist. In conjunction with the outreach team, the authors NH, IM, and JN reached out to the patients and arranged a meeting. NH then met with the patients to provide information about the study and gave them written materials. Patients who met the inclusion criteria were invited to participate in the study. After that, the qualitative interview was carried out or, if the patient preferred, a new date was scheduled for the interview.

All participants participated upon written consent, and the study adhered to the Helsinki Declaration principles. We included patients until the data were saturated, i.e., until no new themes emerged in the qualitative analyses of the interview data.

These two groups of patients were chosen because they are among the most difficult to diagnose, interview, and treat in psychiatry. At the same time, they often only play a minor role in research and interventions targeting schizophrenia. Therefore, there is a critical need to focus on patients with schizophrenia with severely impaired social functioning. Today, we know but little about their everyday lives and how they function outside acute phases.

### Interviews

From October 2021 to June 2022, author NH performed semi-structured, qualitative interviews with each participant following the study’s interview guide. A draft of the interview guide addressing the research questions was created by NH and critically revised by the author group. The interview guide included open and closed questions, addressing the past day in the participant’s life, their self-reported activities, social interactions, expectations, habits, duties, and their wishes for everyday life. The questions were based on previous theoretical studies that had theorized elements of everyday life that might be challenging or different for people with schizophrenia (5). The participants were encouraged to describe their experiences in as much detail as possible through several follow-up questions. The interviews were carried out in settings preferred by the participants. Each participant was interviewed once, which usually lasted between 1 and 1,5 hours. All participants were also assessed on the Global Assessment of Functioning Scale (GAF) (18) and the Personal and Social Performance Scale (PSP). The PSP scale measures the level of impairment in four different domains: socially useful activities, personal and social relationships, self-care, and disturbing and aggressive behavior. The PSP total score, which ranges from 1 to 100, is derived from the levels of impairment in the four domains. A higher total PSP score suggests a higher level of functioning (19).

### Data analysis

All the interviews were recorded verbatim, transcribed, and analyzed by the authors using the qualitative thematic analysis method (20). We used a bottom-up approach in which we coded the data following the principles of thematic analysis, allowing analysis of singular patient’s descriptions and descriptions of experiences across the sample. The researchers familiarized themselves with the data by reading and re-reading the transcribed interviews. In this phase, preliminary ideas for coding the data were generated. Then, codes were coalesced into potential themes, and the themes were reviewed to ensure that they accurately reflected both the coded excerpts and the dataset at large. Finally, key extracts addressing the research questions were selected and embedded in an analytic narrative of the dataset (20, 21).

## Results

We included 17 patients with schizophrenia, of which seven were homeless, and ten were domiciled. See **Table 1** for basic characteristics of the sample.

**Table 1:** Key characteristics of the participants.

	Homeless participants (n=7)	Domiciled participants (n=10)
**Sex (female)**	2	3
**Age (mean (range))**	36 years (22–27)	41 years (35–53)
**Location of interviews**	Shelter: 7	Outpatient clinic: 6
		Participant’s home: 4
**Early retirement benefits (N)**	0	10
**Substance use disorder (N)**	3	3
GAF (mean (range))		
GAF-S	32 (21–39)	38 (31–55)
GAF-F	25 (17–30)	33 (25–40)
PSP (mean (range))		
Total	24.5 (19–32)	40.2 (25–50)
Socially useful activities	3.8 (3–4)	3.4 (3–4)
Personal and social relationships	3.4 (2–4)	2.9 (2–4)
Self-care	3.6 (3–4)	2.6 (1–4)
Disturbing and aggressive behavior	1.6 (0–5)	0.3 (0–3)

GAF, Global Assessment of Functioning; PSP, Personal and Social Performance scale. PSP subscores: absent (0), mild (1), moderate (2), marked (3), severe (4), and very severe (5). PSP total score: A score between 71 and 100 indicates mild functional difficulty, a score between 31 and 70 indicates varying degrees of disability, and a score between 1 and 30 indicates minimal functioning needing intense support and/or supervision.

### Research question 1: What is a typical day like?

Below, we have ordered the findings from our analysis to an ordinary chronology of 24 hours. There were clear differences in a typical day for participants with and without a home.

#### Night

In both groups, most participants kept a certain degree of diurnal rhythm and slept during the night. Yet, several participants from both groups described being awake during the night for various reasons, and more than half of the homeless participants described regularly going days without any sleep:

*“I have always had more energy at night and clearer thoughts. No one is awake outside, so no one can suddenly call me. I am all alone, and that is nice for me (…) It’s easier, for instance, to go for a walk without having to use my brain.”*

*“A year ago, I slept at other people’s places and stayed awake 5–6 days at the time (…) This morning, I got home at 5 am, I think, and then woke up around 2 pm today (…) I am trying to get up before noon and then go brush my teeth, that’s my goal right now.”*

#### Morning

Most participants in both groups described breakfast or morning coffee as a part of their daily routine, either at a specific time or simply as the first thing they did after waking up:

*“I have morning coffee and breakfast. Typically, I drink two cups of coffee, and then I go back to bed (…), then I have lunch when I wake up properly.”*

*“I start the day with morning coffee, and then I take it from there (…) I have no other routines besides that.”*

#### Daytime and evening

Most participants in both groups described doing very little during the day, often being isolated for most of the day, spending time on a hobby, watching TV, or playing video games alone:

*“I only watch TV and smoke weed. That’s all I do every day except when I am shopping for groceries or when I am on the phone with my friend.”*

*“I like sitting at home making creative stuff like Christmas decorations (…) That’s how I pass my day (…) I sit in my own world and relax and have a good time.”*

Most participants in the homeless group described spending a portion of their activities in the presence of others. However, these activities did often not involve much interaction with others. Instead, other people, such as fellow inhabitants in the shelter or people in public space, were just present at the same time as the participant:

*“I spend about an hour reading the news and smoking cigarettes. Then I think I turned on some music, then I just sat in my room and watched TV, smoking cigarettes and drinking coffee most of the day (…). Then, I go down to the common room a few times a day to get a bit of human contact. After 5 pm, we play pool for a few hours, and then we all go to our room again.”*

*“I watch TV a lot. Sometimes, there are other people, and other times, I am the only one interested in what is on TV; that varies. It makes no difference to me. I have no problem watching TV alone, but it’s nice when other people are watching it too.”*

Participants in both groups spent a lot of time engaged in social interactions on social media or interactive online games, and they reported regular interactions and long-lasting relationships with, for example, fellow gamers:

*“I had a good friend in England, a woman at around 45. She died last year of cancer. She was the only good friend I had to talk to. We could talk about anything. She knew I was sick and everything, and I knew about everything in her life. We spoke every day over the computer while playing GTA for almost 20 years.”*

*“I play a lot of Counter-Strike with my mates. That’s fun. There’s a big social aspect to it. The game itself can be very frustrating, but the social aspect makes it worth it (…) A lot of my mates, whom I know from Counter-Strike, I’ve met in real life, so I perceive them as real friends.”*

Some participants reported self-initiated routines or obligations like volunteering to vacuum at the shelter or feeding the birds outside their house. Still, most of their time was spent alone at home or in the shelter.

Some participants described routines surrounding meals like lunch or dinner, but these routines were not anchor points like breakfast or morning coffee. For example, one participant said:

*“Actually, I only ate at half past midnight because I forgot.”*

Below, we offer more detailed descriptions of how two participants, one domiciled and one homeless, spent the last 24 hours.

#### Case 1 – D

Participant D is a 50-year-old man who was diagnosed with schizophrenia 25 years ago. He is living alone in an apartment in Copenhagen. He has a brother he sees a couple of times a month and a father he rarely sees. He has no other close relationships. D has a GAF-Symptom (GAF-S) score of 35 and a GAF-Functioning (GAF-F) score of 30, both reflecting severe illness.

*“I can’t remember if I slept. I think I slept a little bit last night. I woke up early. I do that a lot these days. I either sleep a lot or very little, depending on how tired I am. When I get up, I drink coffee, and sometimes, I drink beer. Then I go to the bakery, where I buy pastries and drink coffee or something like that.”*

*“Sometimes, if I feel inspired, I write poetry and draw and just get into that. Yesterday, I wrote poems, I think. Otherwise, I was just on Facebook, YouTube, email, stuff like that, not much else.”*

*“I go out for a little walk if I have nothing else to do, and I feel bored. I go by the Planetarium and the lakes [i.e., a place in Copenhagen], maybe down to a local park, and I sit there for a while if it’s not too cold. I’ve been there so often just studying people and looking around. I’m used to it. There are so many different types of people. Sometimes, no one really talks to me, and I don’t really talk to them. Sometimes, I end up chatting with people. It differs from time to time.”*

*“I typically go out on my own because I don’t have a lot of friends in Copenhagen. I go to a bar or a pub in the neighborhood. I often talk to random people. That is kind of fun. But that’s most of the social interactions that I have. At my favorite pubs, I know the bartenders and the regulars. I just drink a beer, we say cheers and chat about all kinds of things. Not much more, but it’s nice if you’re used to just sitting at home and feeling bad. Then it’s awesome.”*

#### Case 2 – H

Participant H is a 58-year-old man who was diagnosed with schizophrenia 30 years ago. He is currently staying at a homeless shelter in Copenhagen. Occasionally, he sees his ex-wife and grown-up children, who live in another part of Denmark. He has 4–5 friends whom he sees about once a month. H has a GAF-S score of 39 and a GAF-F score of 28, which both reflect severe illness.

*“I woke up at 7.45. Then I went down to eat breakfast, and then I went back to my room and slept for an hour or two.”*

*“I woke up again and spent an hour or so reading the news and smoking cigarettes. Then I think I put on some music. So, I have just been sitting in my room, watching TV, smoking cigarettes, and drinking coffee for most of the day (…). I look at the national TV stations, I don’t want to pay for other media. I look at different groups on Facebook, where people post stuff I read. I watch a lot of sports. All kinds, I think. That is what I primarily watch when I watch TV – sports and news. Currently, I’m playing an online game called Foundation. It’s like a building game. If it’s a new game that I need to get into and learn, I can spend several days on it and do almost nothing else.”*

*“I go downstairs a couple of times a day to get a bit of human contact. Sometimes, I go downstairs to get a cup of coffee. There is a café where I go and drink coffee. People just sit there and watch TV.”*

*“Nothing is really happening at the shelter until after 5 pm. After 5 pm, we play pool for a couple of hours, and then we all go back to our rooms. So, there is about 2 or 3 hours of social time downstairs. We are a group of 8 to 10 people who play pool. That is kind of what we do together.”*

### Research question 2. What structuring elements do the patients have in their everyday lives?

In our thematic analysis, we identified five themes related to structuring elements in the participants’ everyday lives.

#### Theme 1: Social interactions

All domiciled participants had regular, and sometimes daily, contact with close friends or family via phone or physical meetings.

*“Every day, I, first of all, call my mom. It’s good to have something to look forward to every day. Otherwise, my head would definitely run off, and I would get super paranoid or something.”*

Some homeless participants reported having contact with people close to them, either via phone or in physical meetings. Still, most of the participants had no regular interaction with those close to them. By contrast, they reported that most of their social interactions would be random and with people they only knew superficially, e.g., other inhabitants at the shelter.

*“I socialize with the people around me. I talk to everybody who happens to be here. I’m not going to invite them home or anything (…). I have interactions with the people crossing my path, and that’s that.”*

#### Theme 2: Volunteering

A few participants in both groups described volunteering or having a similar commitment as a structuring element in their everyday life.

*“I help out a little bit here in the shelter. For instance, we have a café, and I like being the one that makes the coffee and hands it out (…). They all come in, and I make them a café latte, espresso, or whatever. I do that 2–3 times a week.”*

*“I go and get newspapers (from the local shop) in the morning. It’s like a job.”*

#### Theme 3: Self-initiated routines

Several participants described routines they liked sticking to, such as going for daily walks or spending time sitting in public places several times a week. This structuring element emerged mainly in the group of domiciled participants. For many of them, this was the only activity they had outside their apartment during the day. Most of them described preferring to go to public places and being around other people, though rarely interacting with them.

*“Down by the shop, there’s a bench. I sit there and drink my morning beer. I talk to people and watch life go by. It’s like watching television.”*

*“You kind of feel like you aren’t alone. You are happy that you aren’t all alone all the time. I tend to socially isolate myself, so getting outside and seeing that there are, in fact, other people out there give me a kind of calmness or certainty. A certainty that life goes on without me. It’s not all about me. It’s nice to get that confirmation because then there isn´t so much pressure on me and my life.”*

#### Theme 4: Exoskeleton

To some degree, the participants staying at a shelter were provided with an externally imposed structure, i.e., an ‘exoskeleton.’ The exoskeleton comprised certain activities and meals during the day. For example, one participant reported that he returns to the shelter every day at 5 pm because this is when dinner is served. However, he did not attend dinner or eat at that time. Another participant said:

*“There are specific times for eating in this shelter. They are my only points of reference: breakfast at 8 am and dinner at 5 pm.”*

By contrast, the domiciled participants had very few or no externally imposed routines (“exoskeleton”) to maintain structures in their everyday lives. Most activities and routines in their everyday life had to be initiated by themselves. However, many of these participants had some exoskeleton in the form of regular visits from case managers from psychiatric services or municipalities.

*“The only person I see regularly apart from my family is J [the contact person from the psychiatric clinic]. She visits me every second week. It’s nice to talk about what’s going on with me and the world around me.”*

#### Theme 5: Pets

Several domiciled participants currently or previously owned pets, and they described being more motivated for tasks related to their pets—e.g., walking the dog or feeding the cat—than for other tasks such as cleaning the house or taking a shower.

*“Well, she (her cat) is a huge advantage. I cannot postpone taking care of her and say, ‘I’ll just wait and do it in the morning.’ I can’t do that with her. If she needs her litter box changed or whatever, I must do it now. Otherwise, she will jump up and meow me in the face. So, if I’m feeling sorry for myself, I will, despite everything, have to get up because of her. Then, when I am up, I might as well stay up.”*

*“It (his bird) somehow also forced me to come home. I couldn’t let it be alone without water. It needs to be refilled at least every third day. So, when I left the apartment, I made sure the food bowls were filled and that it had plenty of water. Then I could be gone for three days.”*

## Discussion

In this study, we explored everyday life in a group of homeless patients staying at a shelter and in a group of domiciled patients. All patients were diagnosed with schizophrenia and had severely impaired social functioning. The descriptions of a typical day in the participants’ lives revealed only few structuring elements for most participants, such as having coffee or breakfast when getting up. None of the participants described much activity during the day, and there was also not much social contact for many of them. However, some of the domiciled participants had regular contact with family or friends. This finding is in line with other studies, which have found that domiciled patients with schizophrenia spend more time at home alone, doing nothing, compared with healthy controls (22–24). In our study, several participants described spending a lot of time thinking, which, from an observational standpoint, could appear to be “doing nothing.” However, for these participants, it did often not feel like “doing nothing,” as having time for thinking was an important part of their life.

Interestingly, the shelters’ routines provided the homeless participants with some structure of their everyday life, e.g., anchor points such as breakfast and dinner at specific times every day, possibilities for social activities in the evening, and opportunities to talk to the staff. In our study, the homeless participants alternated between using these anchor points and withdrawing to their rooms. By contrast, the domiciled participants did not have such an organized structure provided to them. In our sample, several of the domiciled participants had some sort of daily routine, including basic needs (e.g., eating and personal hygiene) and additional activities such as going for a walk each day. Moreover, having pets helped some domiciled participants keep a daily rhythm by requiring them to take care of their pets, and pets were a high priority in their everyday lives.

The finding of a deficient degree of structure in everyday life, especially among the domiciled patients but also the homeless patients, points to an obvious possibility for intervention. As described in the introduction, reducing stress in the patients’ lives can lead to reduced symptomatology, and increased structure is a well-recognized method to reduce stress (7, 8). Assistance in structuring everyday life is often part of treatment in psychiatry, but it is challenging to provide such a structure for outpatients. However, our findings point to an unmet need in this group of patients. One possible way forward could perhaps be to use smartphone apps that could be set up together with a case manager and the phone would provide the necessary notifications.

The homeless participants seemed to have relatively more social contact than the domiciled participants. Yet, the former group also had easier access to others, e.g., other shelter users or staff, around meals or social activities like playing pool or watching TV. By contrast, most domiciled participants had more contact with their family or friends. The social relations of the domiciled participants seemed to be of a closer kind. Still, the relations were fewer and less frequent than the social relations of the homeless participants. Moreover, some domiciled participants were also in some contact with other people, e.g., people they randomly crossed in the street, talked to in a pub, or people they looked at in a park.

However, both groups similarly kept a certain distance from their social environment and regulated the frequency and proximity of their relations to others, e.g., by choosing whether to participate in social activities in the homeless shelter or by observing others in the park. Ellen Corin called this phenomenon in schizophrenia “positive withdrawal” (25). Positive withdrawal is perhaps best viewed as a compensatory strategy by which some patients with schizophrenia balance their need for social interaction with their need to be alone, adopting a generally socially withdrawn position and relating with others in a fairly limited or an anonymous, distanced way, e.g., by watching others in a park or walking through a shopping mall. Corin found that patients who adopted this socially marginalized but not negatively experienced position of positive withdrawal were less likely to be readmitted (26, 27). One could hypothesize that keeping anchor points during the day or other structuring elements, as well as maintaining a flexible distance to the social world, is crucial in negotiating and maintaining a certain balance between the patient’s inner life and the social environment.

The essential role of everydayness in existence is generally overlooked in its complexity and also as a psychotherapeutic target.

## Limitations

Our study is limited to schizophrenia patients with severe social impairment, which is not the case for all patients with schizophrenia. Another limitation is that our study was conducted in a Nordic welfare country with healthcare services free of charge. This context might limit the transferability of our findings to other settings. The sample size is small but within the normal range in a qualitative explorative study.

## Data availability statement

The original contributions presented in the study are included in the article/supplementary material, further inquiries can be directed to the corresponding author/s.

## Ethics statement

Ethical review and approval was not required for the study of human participants in accordance with the local legislation and institutional requirements. The participants provided written informed consent to participate in this study.

## Author contributions

NH: Writing – original draft, Investigation, Formal analysis, Data curation. IM: Writing – review & editing, Supervision, Methodology, Formal analysis, Data curation. AU-P: Writing – review & editing, Supervision, Methodology, Formal analysis, Conceptualization. MH: Writing – review & editing, Supervision, Methodology, Formal analysis, Data curation. JN: Writing – review & editing, Supervision, Methodology, Funding acquisition, Formal analysis, Conceptualization.
